# Quantitative proteomics analysis revealed the potential role of lncRNA Ftx in cardiomyocytes

**DOI:** 10.1186/s12953-022-00201-6

**Published:** 2023-01-05

**Authors:** Xiangfei Sun, Ying Jiang, Qingbao Li, Qi Tan, Mingliang Dong, Bi’e Cai, Di Zhang, Qi Zhao

**Affiliations:** 1 Department of Cardiovascular Surgery, Shandong Provincial Hospital Affiliated to Shandong First Medical University, No. 9677 Jingshi Road, Jinan, 250021 Shandong China; 2 Department of Cardiovascular Surgery, Shandong Provincial Hospital, Cheeloo College of Medicine, Shandong University, Jinan, 250021 Shandong China; 3 Department of Gastroenterology, Shandong Provincial Hospital Affiliated to Shandong First Medical University, No. 324 Jingwu Road, Jinan, 250021 Shandong China; 4 Department of Gastroenterology, Shandong Provincial Hospital, Cheeloo College of Medicine, Shandong University, Shandong 250021 Jinan, China; 5 Health Management Department of Preventive Treatment Center, Affiliated Hospital of Shandong University of Traditional Chinese Medicine, No. 42, Wenhua West Road, Jinan, 250021 Shandong China

**Keywords:** Cardiomyocyte, lncRNA Ftx, Proteomics, Apoptosis, Ferroptosis, Cell cycle

## Abstract

**Objective:**

This study aims to decode the proteomic signature of cardiomyocytes in response to lncRNA Ftx knockdown and overexpression via proteomic analysis, and to study the biological role of lncRNA Ftx in cardiomyocytes.

**Methods:**

The expression level of the lncRNA Ftx in cardiomyocytes cultured in vitro was intervened, and the changes in protein levels in cardiomyocytes were quantitatively detected by liquid chromatography-mass spectrometry. The key molecules and pathways of the lncRNA-Ftx response were further examined by GO, KEGG, and protein interaction analysis.

**Results:**

A total of 2828 proteins are quantified. With a 1.5-fold change threshold, 32 upregulated proteins and 49 downregulated proteins are identified in the lncRNA Ftx overexpression group, while 67 up-regulated proteins and 54 down-regulated proteins are identified in the lncRNA Ftx knockdown group. Functional clustering analysis of differential genes revealed that the lncRNA Ftx is involved in regulating cardiomyocyte apoptosis and ferroptosis and improving cellular energy metabolism. In addition, Hub genes such as ITGB1, HMGA2, STAT3, GSS, and LPCAT3 are regulated downstream by lncRNA Ftx.

**Conclusion:**

This study demonstrates that lncRNA Ftx plays a vital role in cardiomyocytes and may be involved in the occurrence and development of various myocardial diseases. It provides a potential target for clinical protection of the myocardium and reversal of myocardial fibrosis.

**Supplementary Information:**

The online version contains supplementary material available at 10.1186/s12953-022-00201-6.

## Background

The main cellular constituents of the mammalian heart are cardiomyocytes, cardiac fibroblasts, endothelial cells, vascular smooth muscle cells, and immune cells. Among them, cardiomyocytes are the most abundant and occupy 75% of the total myocardial volume [1]. Cardiomyocytes maintain the structure and function of the heart through paracrine, autocrine, and intercellular interactions with other cells [2]. When cardiovascular disease occurs, cardiomyocytes exhibit abnormalities and tend to be replaced by fibroblasts and collagen fibers (i.e., the cardiac remodeling [3]), which is often implicated in heart failure and cardiac death.

Many factors have been shown to be involved in the pathophysiological process of the heart, including the long noncoding RNAs (lncRNAs) which are involved in the epigenetic modification [4]. Studies showed that certain lncRNAs regulate various processes of cardiomyocytes and vascular endothelial cells, including growth, apoptosis, migration and differentiation [4–6], indicating their essential role in the occurrence and development of cardiovascular diseases. Ftx gene is a non-protein-coding gene located on the human X chromosome, which was originally identified in human embryonic stem cells. The Ftx gene encodes the lncRNA Ftx, and its abnormal expression is involved in many congenital and acquired diseases (e.g., tumors and fibrosis [7]). Our group analyzed the cardiomyocyte lncRNA series and found that the trend of lncRNA Ftx in myocardial diseases was still controversial. Drawnel FM et al. [8] showed that lncRNA Ftx changed little in diabetic myocardial injury (GSE62203), whereas Aggarwal P et al. [9] demonstrated that lncRNA Ftx expression was upregulated in the model of myocardial hypertrophy (GSE60291). Previous studies have confirmed that the abnormal expression of lncRNA Ftx played an important role in myocardial ischemia–reperfusion injury [10] and myocardial hypertrophy[11]. However, the specific regulatory mechanism of the lncRNA Ftx on myocardial cells are still under study.

In this study, we intervene the expression level of lncRNA Ftx in cardiomyocytes cultured in vitro and analyze its effects on the downstream proteomics. The aim of this study is to determine the biological effect and regulatory mechanism of lncRNA Ftx on myocardial cells, and further explore its effects on cardiac diseases.

## Materials and methods

### Cell culture

Human cardiomyocyte line AC16 was purchased from the Otwo biotech Inc., and cultured in Roswell Park Memorial Institute (RPMI) 1640 medium supplemented with 10% Fetal Bovine Serum (FBS) under 5% CO_2_ at 37 °C. The culture medium was changed every other day. The cells were digested with trypsin and passaged once the cell fusion rate reached 90%.

### Construction of stable genetically modified cell lines

Based on the manufacturer (Shanghai Genechem, Shanghai, China)’s protocol, the lentivirus-mediated transfection was conducted on (1) the lncRNA Ftx overexpression (Ftx) and its negative control (Ftx-NC) and (2) the lncRNA Ftx interference (Sh-Ftx) and its negative control (Sh-NC). The transfected cells were selected with puromycin (2 μg ml^−1^) and the stability of transfection was validated by real-time qPCR.

### Real-time quantitative PCR assay

Real-time qPCR was performed on the total RNA extracted from the AC16 cells. The primer sequences were shown in Table 1. Beta-actin (ACTB) was used as the internal reference, and the relative expression of the lncRNA Ftx was obtained using the 2^−ΔΔCt^ method.

**Table 1:** Catalog of quantitative qPCR primer sequences

Primers	Sequence (5’-3’)
lncRNA Ftx Forward Primer	GAATGTCCTTGTGAGGCAGTTG
lncRNA Ftx Reverse Primer	TGGTCACTCACATGGATGATCTG
ACTB Forward Primer	TGGCACCCAGCACAATGAA
ACTB Reverse Primer	CTAAGTCATAGTCCGCCTAGAAGCA

### Proteomics analysis

#### Protein extraction and trypsin digestion

Lentivirus-infected AC16 cells were added in the lysis buffer (8 M urea, 1% Protease Inhibitor) and put on the ice for 30 min. After centrifugation at 12,000 × g for 10 min at 4 °C, the supernatant was collected and the protein concentration was determined using a bicinchonic acid (BCA) kit. An equal amount of protein was taken from each sample, and an appropriate amount of standard protein was added. The mixtures were added to the lysis buffer, and the volume of each protein solution was kept the same for all the samples. Then the dithiothreitol (DTT) was added to the protein solutions to make the final concentration 5 mM. The protein solutions were kept at 56 °C to reduce for 30 min. After that, the Iodoacetamide (IAA) was added to make a final concentration 11 mM and the mixtures were incubated in darkness for 15 min at room temperature. The alkylated samples were transferred to an ultrafiltration tube, centrifuged at 12,000 × g for 20 min, replaced for 3 times with the 8 M urea. Then the urea was replaced for 3 times with the replacement buffer. Trypsin was added at a ratio of 1:50 (trypsin to protein, m/m) and the protein was digested overnight. The peptides were recovered after centrifugation at 12,000 × g for 10 min at room temperature, and recovered once more by double distilled (ddH2O). Finally, the two peptide solutions were combined.

#### TMT labeling

The trypsin-digested peptides were desalted by Strata X C18 (Phenomenex) and vacuum-freeze-dried. The peptides were solubilized with 0.5 M TEAB, according to the manufacturer’s protocol for the TMT kit, the labelling reagent were thawed and dissolved with acetonitrile, mixed with the peptides and incubated for 2 h at room temperature. After the labeled peptides were mixed, they were desalted and vacuum-freeze-dried.

#### HPLC fractionation

The peptides were fractionated by the high pH reverse-phase HPLC using the Agilent 300 Extend C18 column (5 μm particles, 4.6 mm ID, 250 mm length). The peptides were first separated into 60 fractions with a gradient of 8–32% acetonitrile at pH 9.0 for 60 min. Then, the peptides were combined into 9 fractions and vacuum-freeze-dried.

#### LC–MS/MS analysis

The tryptic peptides were dissolved in the 0.1% formic acid (mobile phase A), and separated using the NanoElute UHPLC system. Mobile phase A was an aqueous solution containing 0.1% formic acid, while mobile phase B was an acetonitrile solution containing 0.1% formic acid. The gradient settings are: 0–44 min, 6% ~ 24%B; 44–54 min, 24% ~ 36%B; 54–57 min, 36% ~ 80%B; 57–60 min, 80%B, and the flow rate was maintained at 450 nL/min. The peptides separated by the UHPLC system were injected into the Capillary ionization source for ionization and then analyzed by MS/MS in tims-TOF Pro. The ion source voltage applied was 1.65 kV and the peptide parent ions and their secondary fragments were detected and analyzed using TOF. The secondary MS scan range was 100 to 1700 m/z. The data acquisition mode was set as the parallel accumulation-serial fragmentation (PASEF) mode. One primary MS acquisition was followed by 10 PASEF-mode acquisitions of the secondary spectrum with parent ion charge numbers ranged from 0–5. The dynamic exclusion time for the serial MS scans was set to 30 s to avoid duplicated scan of parent ion. The threshold of a significant upregulation was defined as the change of differential expression over 1.5, while the threshold of a significant down-regulation was defined as less than 1/1.5.

#### Bioinformatic analysis

The resulting data were obtained and analyzed by Maxquant (v1.6.6.0). The GO database and KEGG database were used for functional annotation and biological pathway information analysis of the identified proteins. The STRING database (http://www.string-db.org/) was used to construct interaction models between different proteins to visualize and predict the mechanism. Cytoscape (v3.9.1) was used for network analysis, and CytoHubba (v0.1) and MCODE (v2.0.0) plug-ins were used to select the module core protein. The MCC algorithm was used in CytoHubba with the MCODE parameters: Degree Cutoff = 2, Node Score Cutoff = 0.2, K-Core = 2.

### Statistical analysis

SPSS 25.0 and Graphpad Prism 8.0 were used for statistical analysis. The data normality was verified and the variance was verified to be homogeneity. T-test was used for analyze the difference between two groups. One-way ANOVA was used to analyze the difference among multiple groups, and the post-hoc Bonferroni test was used to determine the differences between different groups. A *p*-value < 0.05 was considered statistically significant for all statistical analysis.

## Results

### Efficiency of lentiviral infection

**Fig. 1:**
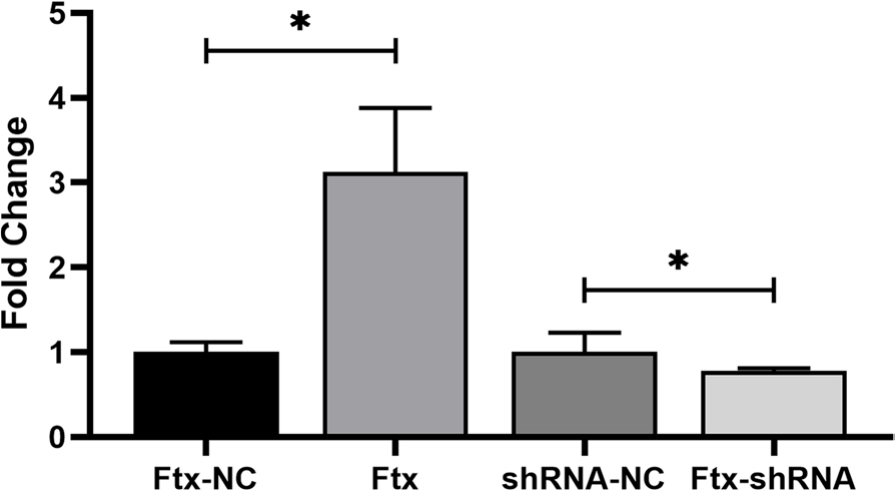
qRT-PCR validation of the lentiviral transfection efficiency. Ftx-NC: the overexpression Ftx negative control lentivirus transfection group. Ftx: the overexpression Ftx lentivirus transfection group, shRNA-NC: the ShRNA negative control lentivirus transfection group, and Ftx-shRNA: the Sh-Ftx lentivirus transfection group. *P < 0.05

AC16 cells are infected with the overexpression lentivirus LV-Ftx, its negative control lentivirus LV-CON007, the ShRNA lentivirus LV-Ftx-RNA, and its negative control lentivirus LV-CON220. The total RNA was collected from each group of cells. Real-time qPCR results confirm the change of Ftx expression caused by the successful transfection of the AC16 cells with the ShRNA and the overexpression lentivirus (Fig. 1).

### Proteome profile of the function loss-and-gain models for AC16 cells

**Fig. 2:**
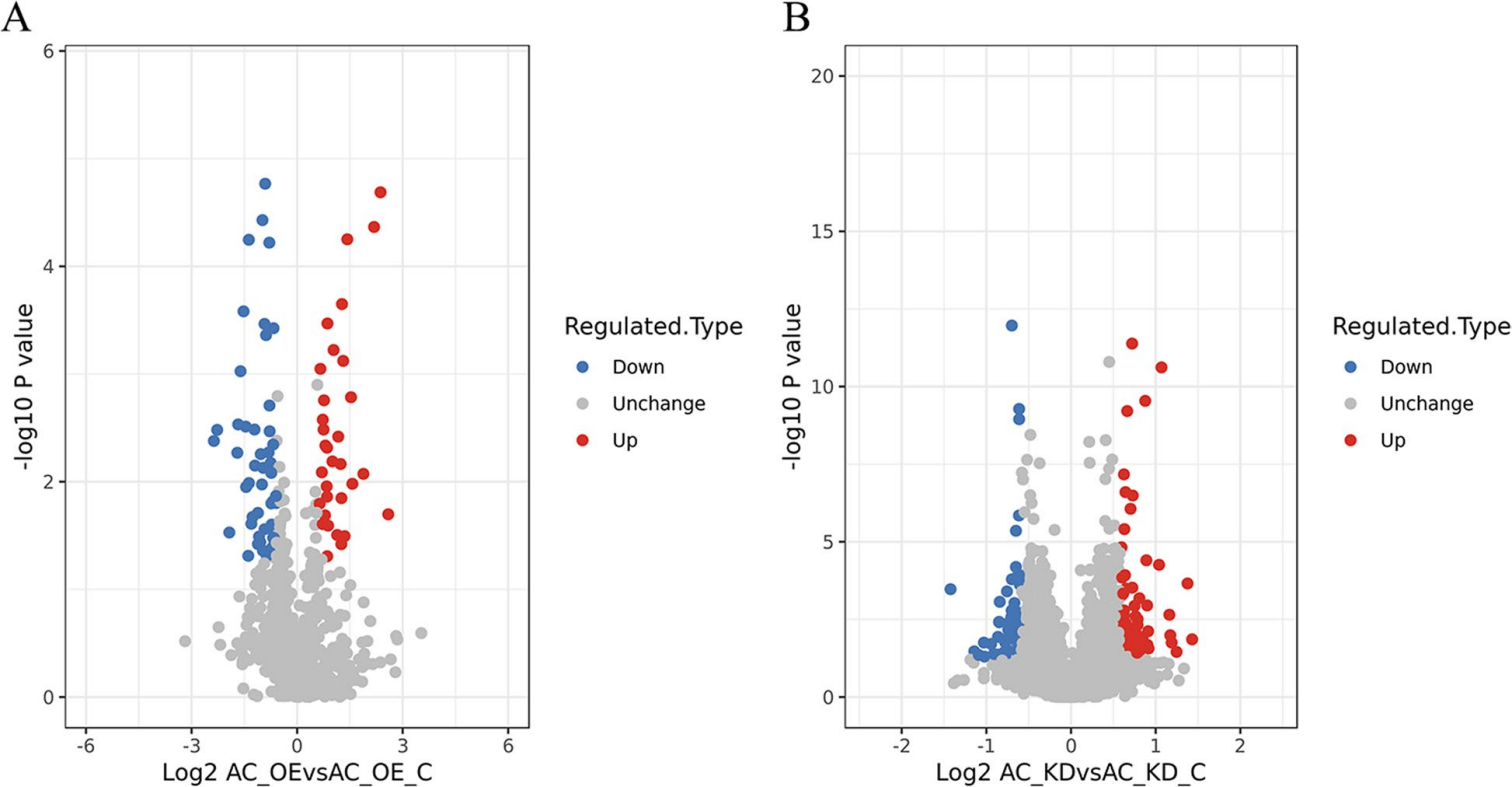
Quantitative volcano plot of differentially expressed proteins. AC_OE: AC16 cells overexpressing Ftx; AC_OE_C: AC16 cells transfected with negative control Ftx-NC; AC_KD: AC16 cells knocked out of Ftx; AC_KD_C: AC16 cells transfected with negative control shRNA-NC

To investigate the effects of the lncRNA Ftx on cardiomyocytes, we performed a quantitative proteomic analysis of the differentially expressed proteins in the function loss-and-gain models for AC16 cells. A total of 4004 proteins were identified by a high-resolution LC–MS/MS analysis, among which 2828 proteins were quantifiable (quantitative information was available for at least one comparable group) (Supplementary Material 1). Compared with the Ftx-NC group, 32 significantly upregulated proteins and 49 significantly downregulated proteins are identified in the Ftx group. Meanwhile, 67 significantly up-regulated proteins and 54 significantly downregulated proteins are identified in the Ftx-Sh group compared with the Sh-NC group. Taking the logarithm of the multiple of protein changing with a base of 2, then map the volcano plot can be obtained (Fig. 2). The 10 most significantly up-regulated and down-regulated proteins among each group are shown in Tables 2 and 3. The results show that the altered expression of the lncRNA Ftx leads to the change in the protein profile of AC16 cells, which may result in the malfunction of cardiomyocytes.

**Table 2:** Top 10 significantly regulated proteins in Ftx-overexpression group

Upregulated protein	Protein description	Downregulated protein	Protein description
Q9UHD2|TBK1	Serine/threonine-protein kinase TBK1	Q92734|TFG	Protein TFG OS = Homo sapiens
Q13620|CUL4B	Cullin-4B OS = Homo sapiens	O15260|SURF4	Surfeit locus protein 4
Q9NZL9|MAT2B	Methionine adenosyltransferase 2 subunit beta	P62136|PPP1CA	Serine/threonine-protein phosphatase PP1-alpha catalytic subunit
Q8WUY1|THEM6	Protein THEM6	Q9NVA2|SEPTIN11	Septin-11 OS = Homo sapiens
O60934|NBN	Nibrin	O14745|SLC9A3R1	Na( +)/H( +) exchange regulatory cofactor NHE-RF1
Q6NUQ1|RINT1	RAD50-interacting protein 1	P61225|RAP2B	Ras-related protein Rap-2b
P2328|PPIB	Peptidyl-prolyl cis–trans isomerase B	Q9Y230|RUVBL2	RuvB-like 2
O43819|SCO2	Protein SCO2 homolog, mitochondrial	Q9NX63|CHCHD3	MICOS complex subunit MIC19
Q15393|SF3B3	Splicing factor 3B subunit 3	P51665|PSMD7	26S proteasome non-ATPase regulatory subunit 7
Q10570|CPSF1	Cleavage and polyadenylation specificity factor subunit 1	P60033|CD81	CD81 antigen

**Table 3:** Top 10 significantly regulated proteins in Ftx-knockdown group

Upregulated protein	Protein description	Downregulated protein	Protein description
P38432|COIL	Coilin	Q86V21|AACS	Acetoacetyl-CoA synthetase
P55283|CDH4	Cadherin-4	Q9Y673|ALG5	Dolichyl-phosphate beta-glucosyltransferase
Q1ED39|KNOP1	Lysine-rich nucleolar protein 1	P31350|RRM2	Ribonucleoside-diphosphate reductase subunit M2
P52926|HMGA2	High mobility group protein HMGI-C	P31751|AKT2	RAC-beta serine-/threonine-protein kinase
P19971|TYMP	Thymidine phosphorylase	Q9NNW5|WDR6	WD repeat-containing protein 6
P42166|TMPO	Lamina-associated polypeptide 2, isoform alpha	Q9BQE3|TUBA1C	Tubulin alpha-1C chain
P1104|LAMC1	Laminin subunit gamma-1	Q96ME1|FBXL18	F-box/LRR-repeat protein 18
P17096|HMGA1	High mobility group protein HMG-I/HMG-Y	O95757|HSPA4L	Heat shock 70 kDa protein 4L
Q9UJZ1|STOML2	Stomatin-like protein 2, mitochondrial	P52566|ARHGDIB	Rho GDP-dissociation inhibitor 2
Q5T8P6|RBM26	RNA-binding protein 26	P31689|DNAJA1	DnaJ homolog subfamily A member 1

### Functional enrichment and clustering analysis of differentially expressed proteins

**Fig. 3:**
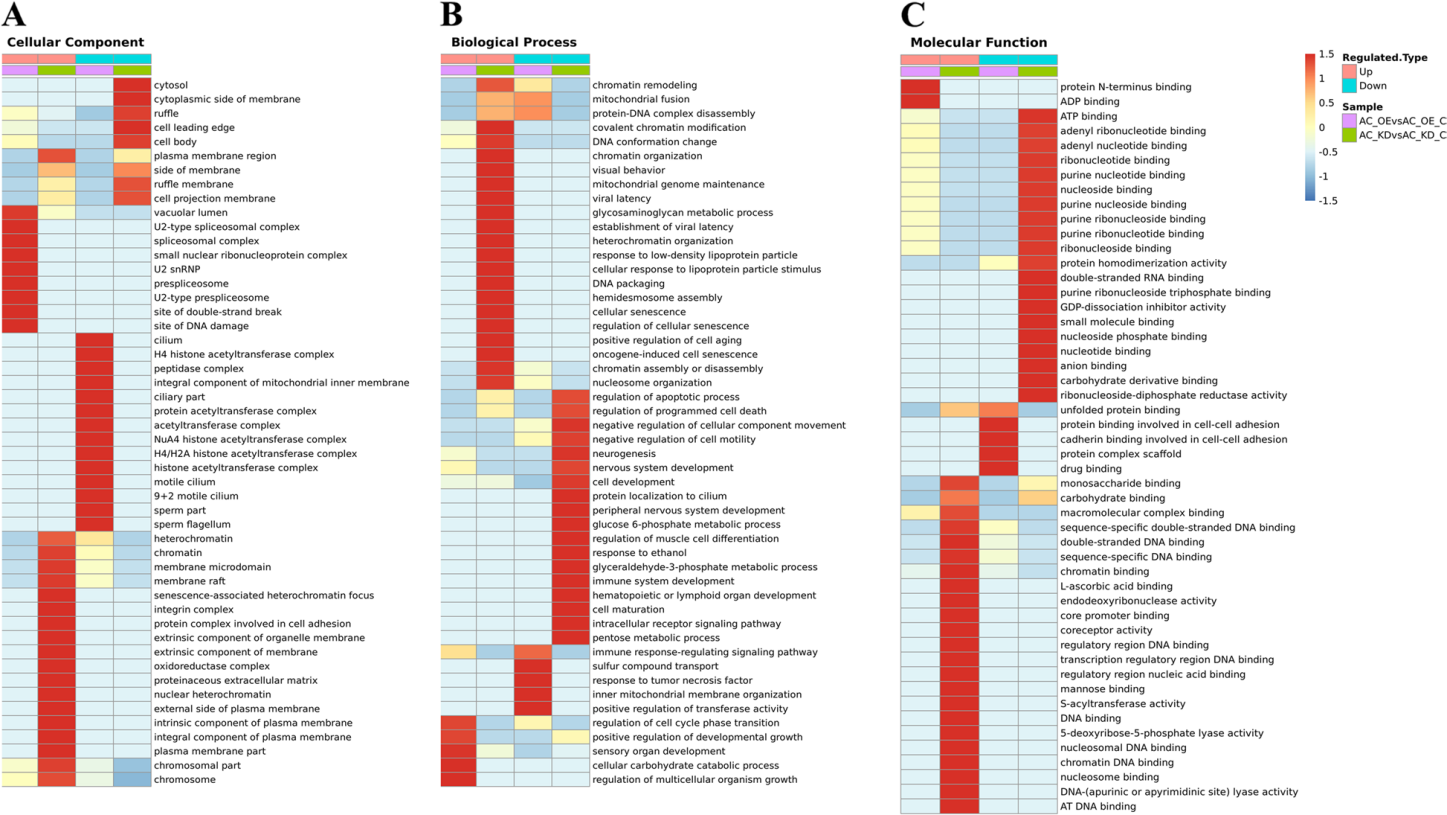
GO enrichment clustering analysis of differentially quantified proteins. **A** Cellular components; **B** Biological processes; **C** Molecular functions. AC_OE: AC16 cells overexpressing Ftx; AC_OE_C: AC16 cells transfected with negative control Ftx-NC; AC_KD: AC16 cells knocked out of Ftx; AC_KD_C: AC16 cells transfected with negative control shRNA-NC

The enrichment and clustering analysis were performed to identify the related biological function and the nature of differentially expressed proteins by lncRNA Ftx overexpression or knockdown. GO functional enrichment analysis shows (Supplementary Material 2, 3) that the differentially expressed proteins after the lncRNA Ftx knockdown or overexpression exists mainly in organelle, cell, membrane-enclosed lumen and other cell components. They perform molecular functions such as binding and catalysis, and are mainly involved in the cellular process, metabolic process, single-organism process, and biological regulation. Cluster analysis based on GO enrichment further reveals (Fig. 3) that the lncRNA Ftx knockdown leads some cellular components, such as heterochromatin and chromatin, to be enriched by upregulated proteins, while leads other components, such as cytosol, cell leading edge, and ruffle, to be enriched by downregulated proteins. The overexpression of lncRNA Ftx leads some components, such as the U2-type prespliceosome, site of DNA damage, and site of double-strand break, to be enriched by upregulated proteins, while leads other components, such as protein complex scaffold, unfolded protein binding, drug binding, and cadherin binding involved in cell–cell adhesion, to be enriched in downregulated proteins. In the biological process, knockdown of LncRNA Ftx leads the positive regulation of cell aging and the oncogene-induced cell senescence to be enriched by upregulated proteins, while leads the negative regulation of cellular component movement, cell development, and negative regulation of cell motility to be enriched by downregulated proteins. Meanwhile, when lncRNA Ftx is overexpressed, the upregulated proteins are mainly associated with the positive regulation of developmental growth, regulation of cell cycle phase transition, regulation of cell cycle process, and cell proliferation, while downregulated proteins are mainly associated with the inner mitochondrial membrane organization, mitochondrial fusion, protein-DNA complex disassembly, and response to the tumor necrosis factor. When lncRNA Ftx is knockdown, upregulated proteins enrich in the chromatin DNA binding and nucleosome binding, while downregulated proteins enrich in the carbohydrate derivative. When lncRNA Ftx is overexpressed, upregulated proteins enrich in the protein N-terminus binding and downregulated proteins enrich in the sperm part.

**Fig. 4:**
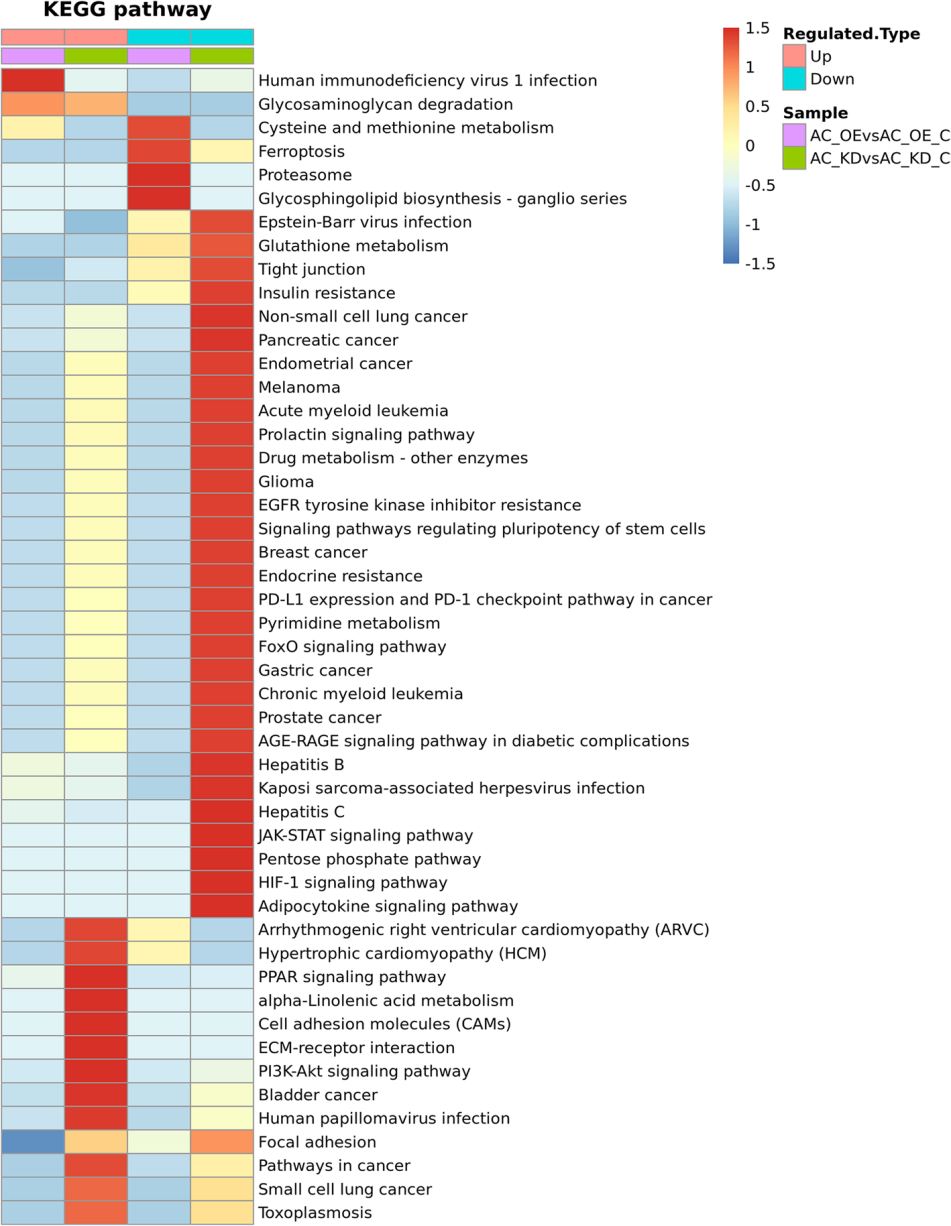
Cluster analysis of the KEGG pathway for differentially quantified proteins. AC_OE: AC16 cells overexpressing Ftx; AC_OE_C: AC16 cells transfected with negative control Ftx-NC; AC_KD: AC16 cells knocked out of Ftx; AC_KD_C: AC16 cells transfected with negative control shRNA-NC

We then further performed a pathway enrichment-based clustering analysis of the lncRNA Ftx-responsive proteome employing the pathways identified by the KEGG analysis (Fig. 4, Supplementary Material 4, 5). It shows that the protein expression involved in cell adhesion molecules (CAMs), PI3K-Akt, and PPAR signaling pathways are enriched by upregulated proteins after lncRNA Ftx knockdown, whereas JAK-STAT and HIF-1 signaling pathways are enriched by downregulated proteins. Meanwhile, the overexpression of the LncRNA Ftx leads the Human immunodeficiency virus 1 infection and Glycosaminoglycan degradation pathways to be enriched by upregulated proteins, while leads the pathways including Proteasome and Ferroptosis to be significantly downregulated.

**Fig. 5:**
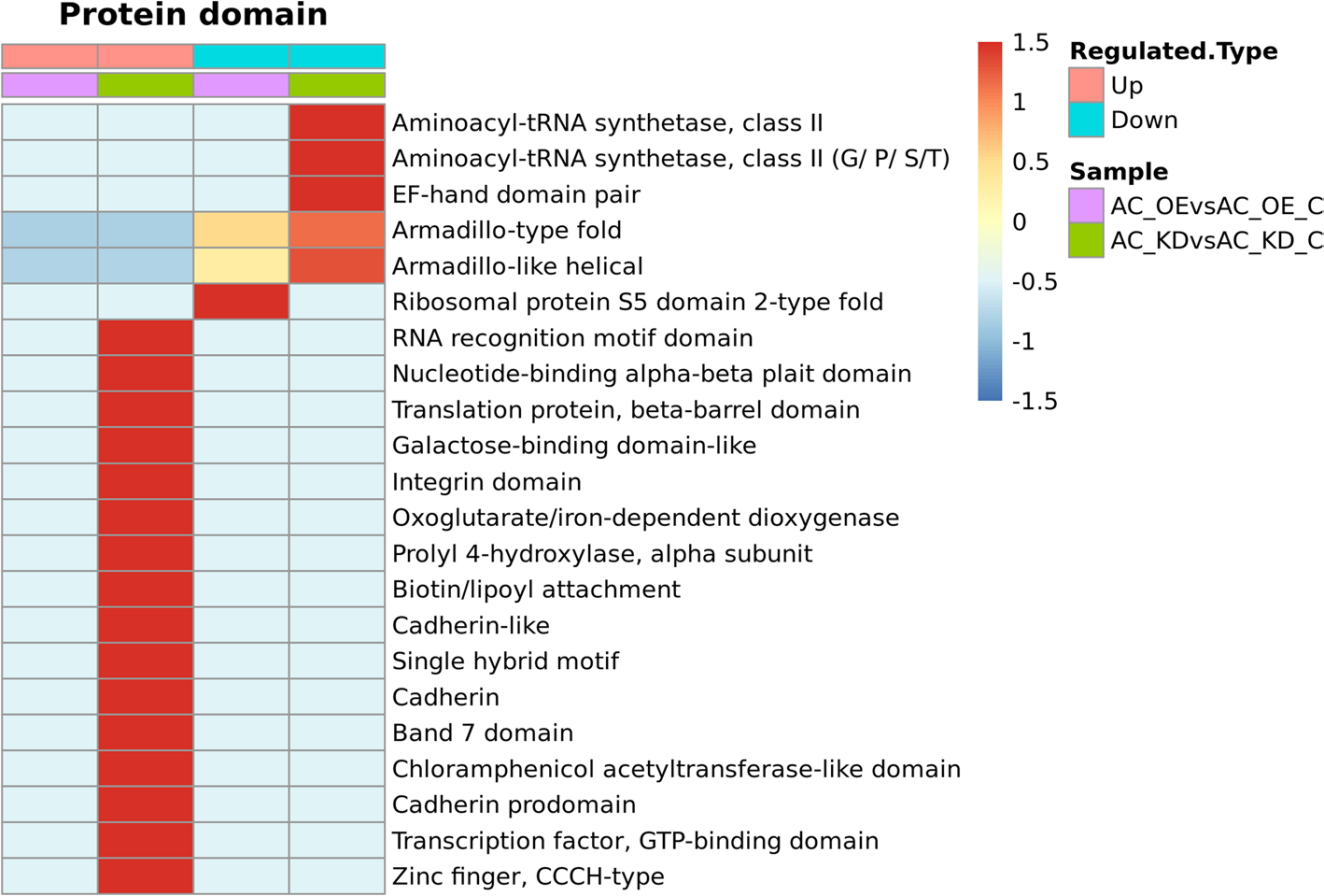
Protein domain-based clustering analysis of differentially quantified proteins. AC_OE: AC16 cells overexpressing Ftx; AC_OE_C: AC16 cells transfected with negative control Ftx-NC; AC_KD: AC16 cells knocked out of Ftx; AC_KD_C: AC16 cells transfected with negative control shRNA-NC

Finally, to define the domain characteristics of the differentially quantified proteins induced by lncRNA Ftx knockdown and overexpression, we performed the analysis of the domain enrichment based on the previous domain analysis (Fig. 5, Supplementary Material 6, 7). The results shows that the upregulated proteins affected by lncRNA Ftx knockdown contain the Chloramphenicol acetyltransferase-like domain, Cadherin prodomain, Band 7 domain, and Cadherin, while the downregulated proteins contain the Aminoacyl-tRNA synthetase and class II domain. After the overexpression of the lncRNA Ftx, the downregulated proteins contain the Ribosomal protein S5 domain 2-type fold.

### Protein–protein interaction network analysis

**Fig. 6:**
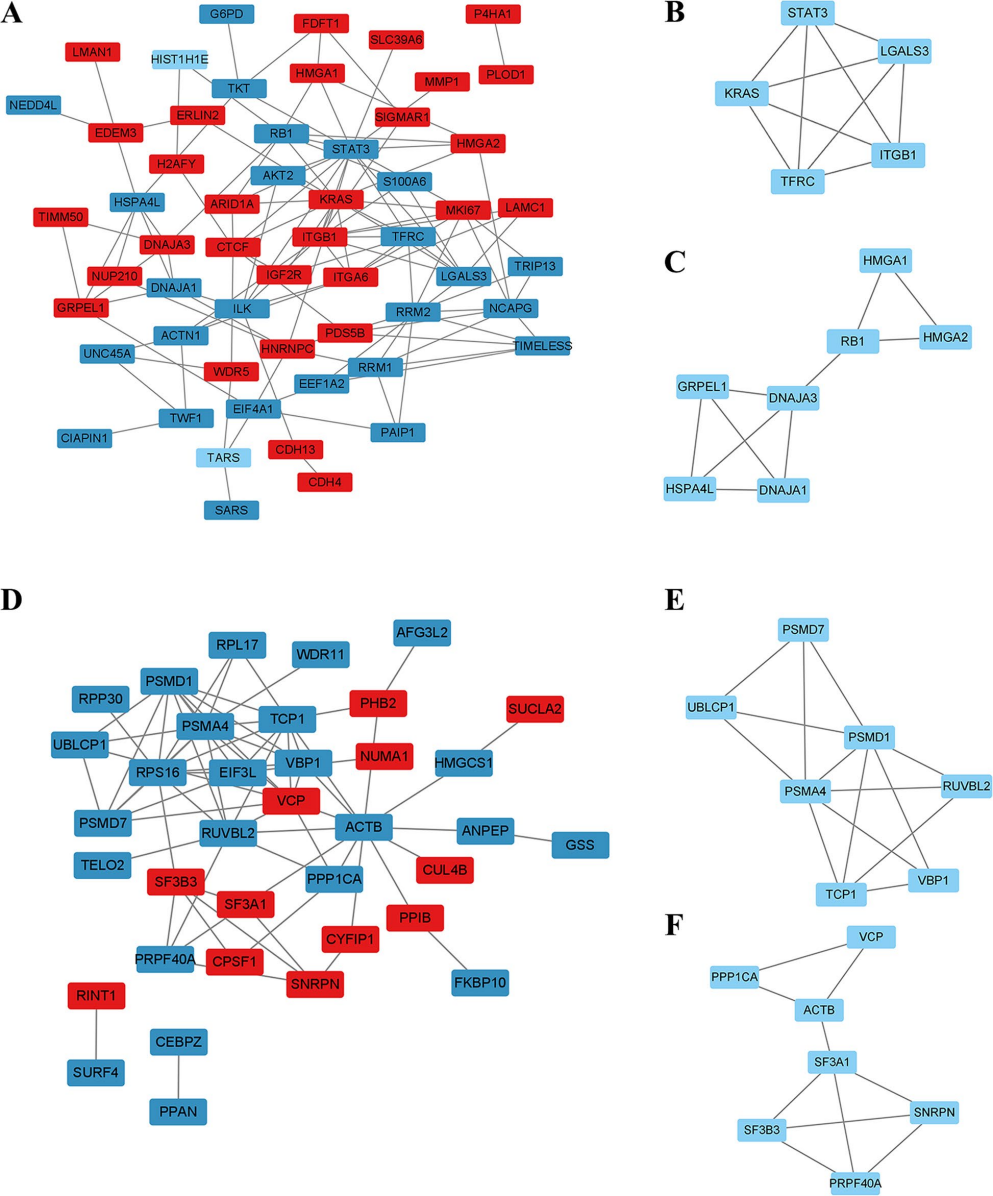
Protein interaction network analysis. **A** Protein interactions network diagram (red represents upregulation, blue represents downregulation); **B** Protein interactions core module

The STRING database was used to visualize the reciprocal network between differentially expressed proteins (Fig. 6). A total of 37 proteins are matched in the overexpressed lncRNA Ftx group, with an interaction network p-value of 8.84e-11 and an average interaction score of 3.78. Two core modules are identified by MCODE, with one containing PSMA4, PSMD7, PSMD1, RUVBL2, VBP1, TCP1, UBLCP1 and the other one containing ACTB, VCP. The top 10 Hub genes identified by the MCC-algorithm-based CytoHubba plug-in are PSMD1, PSMA4, TCP1, VCP, RPS16, RUVBL2, VBP1, PSMD7, ACTB, UBLCP1. 70 proteins are matched in the knockdown lncRNA Ftx group, with an interaction network p-value of 6.33e-15, and an average interaction score of 3.93. The 2 core modules identified by MCODE contain TFRC, KRAS, STAT3, LGALS3, ITGB1 and HMGA2, HMGA1, RB1, and the top 10 Hub genes are KRAS, STAT3, ITGB1, TFRC, LGALS3, RB1, ITGA6, MKI67, RRM2, NCAPG.

## Discussion

After injury, the heart inevitably undergoes fibrosis which leads to heart failure. The development of injury-induced myocardial fibrosis is resulted from the interaction of multiple factors, including the matrix structural remodeling and the cell functional change [12]. As cardiomyocytes plays an important role in the heart functioning, a further study on cardiomyocytes can lead to better understanding of the mechanism of the heart disease development. Besides, it also provides an important target molecule for clinical gene therapy of heart diseases.

Recently, non-coding RNAs have become a major focus of biological research. Longer than 200 nucleotides and without protein-coding potential, the long noncoding RNAs (lncRNAs) are highly abundant in the human body [13]. As a newly found epigenetic regulatory molecule, the lncRNAs attract a lot of attention in the field of cardiovascular disease research [14]. It shows that certain lncRNAs are regulated in acute myocardial infarction (e.g. Novlnc6 [4]) and heart failure (e.g. Mhrt [6]). In addition, lncRNAs demonstrate an ability to control the cardiomyocyte hypertrophy, mitochondrial function and apoptosis. In the vascular system, the endothelin-expressing lncRNAs (e.g.MALAT1 [15]) shows to regulate growth and function of the vascular, while the smooth muscle-expressing lncRNAs and the migration/differentiation-related lncRNAs enriching in endothelial cells prove to control the contraction of the smooth muscle cells [16]. These findings suggest that lncRNAs play a significant role in regulating the development of cardiovascular disease.

The Ftx gene is a non-protein-coding gene located on the human X chromosome. Its DNA sequence encodes nine introns, seven of which are transcribed to RNA fragments and then linked to be lncRNA Ftx (long non-coding RNA Ftx, Ftx) [7]. LncRNA Ftx consists of approximately 2300 nucleotides. In the past, studies on the function of Ftx focused on its involvement in the development of congenital diseases [17]. Recently, studies turn to the regulatory role of Ftx in acquired diseases. Studies demonstrate that lncRNA Ftx induces the progression of liver cancer, colon cancer, kidney cancer, and liver fibrosis [18, 19]. For cardiovascular diseases, Long B et al. shows that the lncRNA Ftx is significantly downregulated in the ischemia–reperfusion injured and hydrogen peroxide-treated myocardial tissue, and the overexpression of lncRNA Ftx attenuates the apoptosis induced by hydrogen peroxide in cardiomyocyte. This function may be associated with the regulation of Bcl212 expression by lncRNA Ftx-mediated miR-29b-1-5p [20]. Recent studies find that overexpression of lncRNA Ftx can upregulate Fmr1 by sponging miR-410-3p (fragile X mental retardation 1) to induce cell proliferation, inhibit apoptosis and oxidative stress, which alleviates the cardiomyocyte injury induced by hypoxia/reoxygenation [10]. Yang X et al. uses arginine II to induce hypertrophy of neonatal mouse cardiomyocyte in vitro and demonstrates that the expression of lncRNA Ftx is significantly downregulated. While the overexpression of lncRNA Ftx significantly reduces the apoptosis, myocardial contractility, and the expression of some key molecules such as c-Jun, A-type natriuretic peptide (ANP) and B-type natriuretic peptide (B). A further study confirms that lncRNA Ftx played a role in reducing myocardial hypertrophy by sponging miRNA-22 to regulate the PTEN/PI3K/Akt signaling pathway [11].

Previous findings confirm the differential expression levels of lncRNA Ftx in cardiac diseases, suggesting its regulatory role in the cardiac disease development, but the specific regulatory effects and mechanisms have not been fully studied. To investigate the effects and potential molecular mechanisms of lncRNA Ftx on cardiomyocytes, this work establish the lncRNA Ftx function gain-and-loss model in the cardiomyocyte AC16 cell line. The overall proteome of cardiomyocyte in response to lncRNA Ftx knockdown and overexpression is systematically characterize with the quantitative proteomics and bioinformatics analysis.

Combined with the functional clustering analysis of the selected Hub genes and differential proteins, we find that knockdown of lncRNA Ftx upregulates the proteins associated with cell aging, fibrogenic differentiation, and apoptosis, while downregulates the proteins associated with cell cycle, cell proliferation, and anti-apoptosis to a certain extend. These alterations are dominated by the Hub genes ITGB1, TFRC, and RB1. In terms of pathways, the downregulation of the lncRNA Ftx upregulates the activation of the CAMs, PI3K-Akt signaling pathway, and PPAR signaling pathway, while inhibits the activation of JAK-STAT signaling pathway. On the other hand, the overexpression of the lncRNA Ftx upregulates the proteins associated with positive regulation of growth and cell cycle, and downregulates the iron death-related pathways, evidenced by the altered expression levels of several Hub genes, including VCP, PSMD1, 4,7, GSS and LPCAT3. The difference in protein expression of AC16 cells is associated with the LncRNA Ftx overexpression and knockdown, indicating the lncRNA Ftx is involved in altering myocardial function, and making it a potential target for disease occurrence, development and treatment.

The normal regulation of the cell cycle is essential for cell proliferation, differentiation, and apoptosis. Lack of regenerative capacity, cardiomyocytes are replaced by collagen fibers after dying due to inflammation or injury, which eventually leads to myocardial remodeling. Therefore, apoptosis and ageing of the cardiomyocyte plays an essential role in the myocardial disease development, and the inhibition of apoptosis in myocardial ischemic disease shows to mitigate this process [21, 22]. In the lncRNA Ftx dysfunction model, we identify multiple proteins involved in apoptosis, evidenced by the upregulation of ITGB1, HMGA2, KRAS and the downregulation of STAT3, RB1, LGALS3.

ITGB1 (Integrin β1), a member of the Integrin family, acts as an extracellular matrix receptor that regulate cell–matrix interactions, cell proliferation, and epithelial-mesenchymal transformation [23]. ITGB1 have impacts on the cardiovascular system in several aspects, including myocardial function and differentiation [24]. Transient episodes of myocardial ischemia promote the proliferation of endothelial cells and the formation of small arteries throughout the myocardium in response to the myocardial infarction, where the ITGB1 is involved [25]. Some study shows that the cardiomyocyte-specific knockdown of ITGB1 leads to the development of myocardial fibrosis and heart failure [26]. However, the proteomic analysis in this study show that the knockdown of lncRNA Ftx resulted in the upregulation of ITGB1 expression, and more studies will be needed to reveal the effects of the lncRNA Ftx on the myocardium.

HMGA2 (High mobility group protein AT-hook 2) belongs to the high mobility A genome. As a structural transcription factor, HMGA2 is important for cell growth and differentiation and is involved in the epithelial mesenchymal transformation [27]. A study confirms that HMGA2 plays a crucial role in cardiogenesis and remodeling [28]. Wong, L. L. et al. shows that targeting the 3'-UTR of HMGA2 could inhibit apoptosis and protect cardiomyocyte from ischemic injury [29]. HMGA2 induces the apoptosis by upregulating cleaved Caspase 3 through the DNA damage pathway, associated with the upregulation of cleaved Caspase 9, p53, Bax, and the downregulation of Bcl2, Apaf1. However, another study in a mouse model of myocardial remodeling demonstrates that the cardiac-specific expression of HMGA2 reduces myocardial fibrosis and improves cardiac function by activating the PPAR pathway [30]. Our results also show the concomitant activation of the PPAR pathway in the presence of upregulated HMGA2. Therefore, the specific effects of the upregulated HMGA2 protein after lncRNA Ftx knockdown need to be further investigated.

STAT3 is an important factor of the signaling pathway, and its downstream target genes are involved in the regulation of cell differentiation, proliferation, apoptosis, angiogenesis, metabolism and immune response, etc. The protective effects of STAT3 on cardiomyocytes are reflected by the anti-apoptosis and the energy generation. On the one hand, STAT3 helps the cardiomyocyte survive by upregulating the expression of anti-apoptotic genes Bcl-xL and Bcl2[31], and block the TNF-α pro-apoptotic channel [32]. On the other hand, STAT3 demonstrates to present in the mitochondria of cardiomyocyte, which regulates the activity of the type I complexes and oxygen consumption, and participates in energy production [33]. In addition, recent studies demonstrates that the JAK/STAT3 signaling pathway plays a crucial role in the induction, maintenance, and differentiation of the multipotential stem cells [34]. Further studies on STAT3 will benefits the cardiac regenerative therapy.

In addition, we notice a programmed cell death modulated by lncRNA Ftx which is distinct from apoptosis—ferroptosis. Overexpression of the lncRNA Ftx in cardiomyocyte shows to downregulates the ferroptosis pathway-related proteins (GSS and LPCAT3), indicating that the overexpression of lncRNA Ftx may play a protective role in cardiac disease by inhibiting ferroptosis in the cardiomyocyte.

Ferroptosis is a newly found mode of programmed cell death characterized by iron overload, reactive oxygen species (ROS) accumulation, or lipid peroxidation. Distinct from apoptosis, pyroptosis, autophagy and necrosis, it is first identified by Brent R. Stockwell’s laboratory [35, 36]. The cells with ferroptosis exhibit morphological loss of membrane integrity, accompanied by nuclei swelling, mitochondria crinkling, cristae reduction or absence, and outer membranes fragmentation [37]. Ferroptosis shows to be associated with a variety of diseases such as tumor, degenerative disease (Alzheimer's disease, Huntington's chorea, Parkinson's syndrome), and renal failure [38]. Ferroptosis is also involved in the development of many cardiac diseases, including myocardial ischemia–reperfusion injury, myocardial hypertrophy, diabetic heart disease, and doxorubicin-induced cardiotoxicity [39, 40].

Ferroptosis is closely related to many biological processes such as iron metabolism, glutathione (GSH) metabolism and lipid peroxidation. Therefore, the molecules involved in these metabolic pathways can affect the level of ferroptosis in cells [41]. GSS (Glutathione synthetase) is involved in the GSH anabolism and responds rapidly to the increased GSH demand. Glutathione peroxidase 4 (CPX4), an important negative regulator of ferroptosis, shows to play a biological role by regulating the GSS/GSR complexes [42]. LPCAT3 (lyso-phosphatidylcholine acyltransferase-3) is a major lyso-PL acyltransferases (LPLAT) isomer exhibiting a strong specificity to the polyunsaturated fatty acid (PUFA) [43]. Both of the proteins shows to be negatively regulated by the lncRNA Ftx, though further experimental evidence is needed to correlate them to the ferroptosis inhibition.

In summary, this study, for the first time, provides the data on the comprehensive changes in the quantitative proteomic profile of cardiomyocytes after lncRNA Ftx knockdown and overexpression by constructing the lncRNA Ftx function gain-and-loss model for the AC16 cells, with quantitative proteomics and bioinformatics analysis. The protein annotation, enrichment and clustering analysis reveal the properties of the differentially quantified proteins identified from the quantitative proteomic data. Potential Hub genes are selected by the protein interaction network analysis. Our results show that the lncRNA Ftx regulates the apoptosis and ferroptosis in cardiomyocytes and improves the cellular energy metabolism. LncRNA Ftx is involved in expression changes of several proteins such as ITGB1, HMGA2, STAT3, GSS and LPCAT3. It demonstrates to play a vital role in the occurrence and progression of myocardial diseases such as ischemia–reperfusion injury, myocardial hypertrophy, and myocardial fibrosis, thus provides a promising target for the protection of the myocardium and the reversal of myocardial fibrosis.

## Supplementary Information

**Additional file 1.** 

**Additional file 2.** 

**Additional file 3.** 

**Additional file 4.** 

**Additional file 5.** 

**Additional file 6.** 

**Additional file 7.** 

## Data Availability

The datasets generated during and analyses during the current study are available in the figshare (https://doi.org/10.6084/m9.figshare.19807657.v1).
